# Building the Evidence for Hospital Mobility

**DOI:** 10.1093/geroni/igaa057.3205

**Published:** 2020-12-16

## Abstract

Low mobility in the hospital, defined as mobility limited to bed rest or bed to chair transfers, is associated with high rates of functional decline, nursing home placement, and death even after adjusting for illness severity and comorbidity. This lecture will describe the gradual of building of evidence for both the adverse outcomes and potential solutions at both an individual and a health system level to address the challenge of low mobility.

